# Ruptured Rudimentary Horn Pregnancy: Delayed Diagnosis

**DOI:** 10.7759/cureus.15873

**Published:** 2021-06-23

**Authors:** Maninder K Ghotra, Bharti Joshi, Shinjini Bhutani

**Affiliations:** 1 Obstetrics and Gynaecology, Post Graduate Institute of Medical Education & Research (PGIMER) Chandigarh, Chandigarh, IND

**Keywords:** rudimentary horn pregnancy, ruptured, developmental anomalies, hemodynamic instability, severe anemia

## Abstract

Developmental anomalies of the genital tract result from defective fusion and absorption of various parts of Mullerian ducts in fetal life. Rudimentary horn pregnancy is a rare occurrence of one in 76,000 and one in 160,000. We present a case of a 24-year-old primigravida with ruptured rudimentary horn pregnancy initially managed in the line of an intrauterine pregnancy with severe anemia. Hemodynamic instability made us suspect ruptured rudimentary horn pregnancy and lifesaving laparotomy was performed for the same. A 1.5-liter hemoperitoneum was encountered with a right ruptured rudimentary horn. Multiple adhesions were present with necrotic tissue adherent and clumped together as tubo ovarian mass. Resection of the rudimentary horn was performed. We report this case to emphasize the need to consider rare uterine anomalies as a possibility in patients presenting with acute abdomen in early pregnancy. Obstetricians should consider these rare entities in the differential diagnosis to provide efficient management of these cases.

## Introduction

A ruptured rudimentary horn is a life-threatening obstetrical emergency where the diagnosis is usually either missed or delayed. A rudimentary horn is a developmental anomaly of fetus. In 1979, Buttram and Gibbon classified Mullerian anomalies, which were modified by the American Fertility Society (1988) later, in which unicornuate uterus is classified as type 2 Mullerian anomaly with further classification of the horn as communicating, non-communicating, no cavity, and no horn. According to the ESHRE/ESGE classification system (2013), our patient is classified under U4a (unicornuate uterus with communicating horn). These conditions are associated with various complications such as endometriosis, hematometra, infertility, urinary tract anomalies, abortions, preterm deliveries which increase morbidity but rupture during pregnancy is life-threatening to the patient. We report a case of ruptured rudimentary horn pregnancy at 11 weeks period of gestation, which was initially diagnosed as severe anemia with pregnancy.

## Case presentation

**Figure 1:**
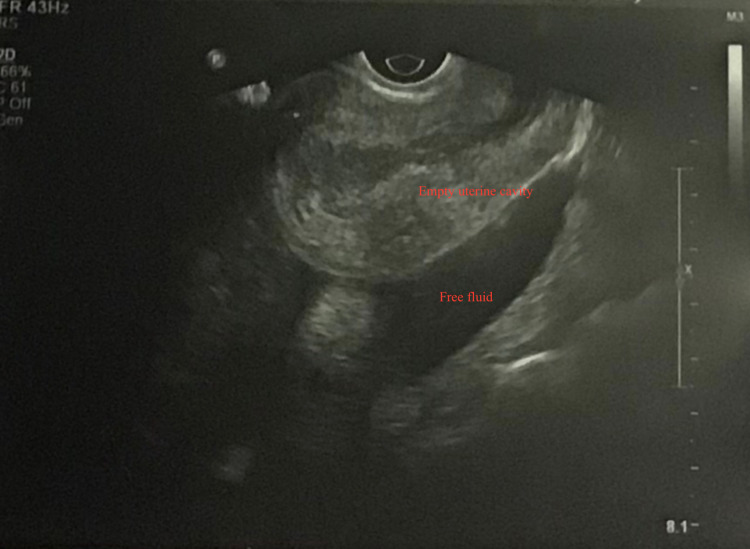
Ultrasound image showing gross hemoperitoneum with the empty uterine cavity

**Figure 2:**
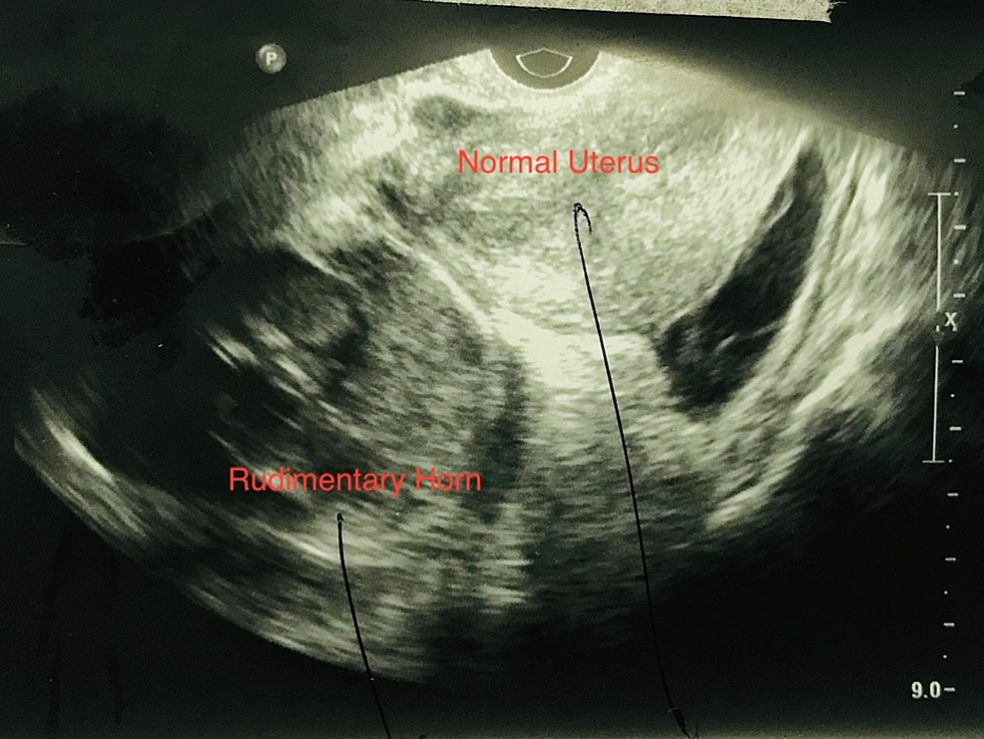
Ultrasound image showing normal uterus with ruptured rudimentary horn with a fetus surrounded by myometrium of the rudimentary horn

A 24-year-old primigravida was referred to our institute at 11+5 weeks of pregnancy in a hemodynamically unstable condition with severe anemia and hemoperitoneum as a suspected case of ruptured ectopic pregnancy. There was no significant past medical or surgical history. She had had achieved menarche at 14 years of age and had normal menstrual cycles with no associated dysmenorrhea. She was admitted for four days at a multi-specialty hospital (at 11+1 weeks) with complaints of weakness and easy fatigability and was diagnosed as severe anemia with pregnancy. Transabdominal ultrasound revealed a single viable intrauterine fetus corresponding to 9+5 weeks' gestation (before admission), and she had received blood transfusion (hemoglobin - 5.5 g/dL). She had a repeat ultrasound done after two weeks ( 11+5 weeks) following an episode of abdominal pain with vomiting (during admission and post blood transfusion), and which revealed a suspicion of 11 weeks ectopic pregnancy with fetal heart tones along with gross hemoperitoneum, confirmed on flank paracentesis. She was referred in view of hemodynamic instability and severe anemia with a provisional diagnosis of ruptured ectopic pregnancy. On admission, she was pale and afebrile and tachycardiac (130 bpm) with a blood pressure of 100/60 mmHg. On examination, her abdomen was uniformly distended with tenderness and rigidity with local examination showing no bleeding and on vaginal examination cervical motion tenderness was found (+Frenkel sign) with right forniceal tenderness and fullness, uterine size was not appreciated. Her laboratory bloodwork evidenced hemoglobin of 8.3 g/dL and platelet count of 160,000/microliter with a normal coagulation profile. On ultrasound, there was evidence of hemoperitoneum (Figure 1) with live pregnancy of 10 weeks surrounded by myometrium around it in the right adnexa with a thickened endometrium and empty uterine cavity (Figure 2).

 

**Figure 3:**
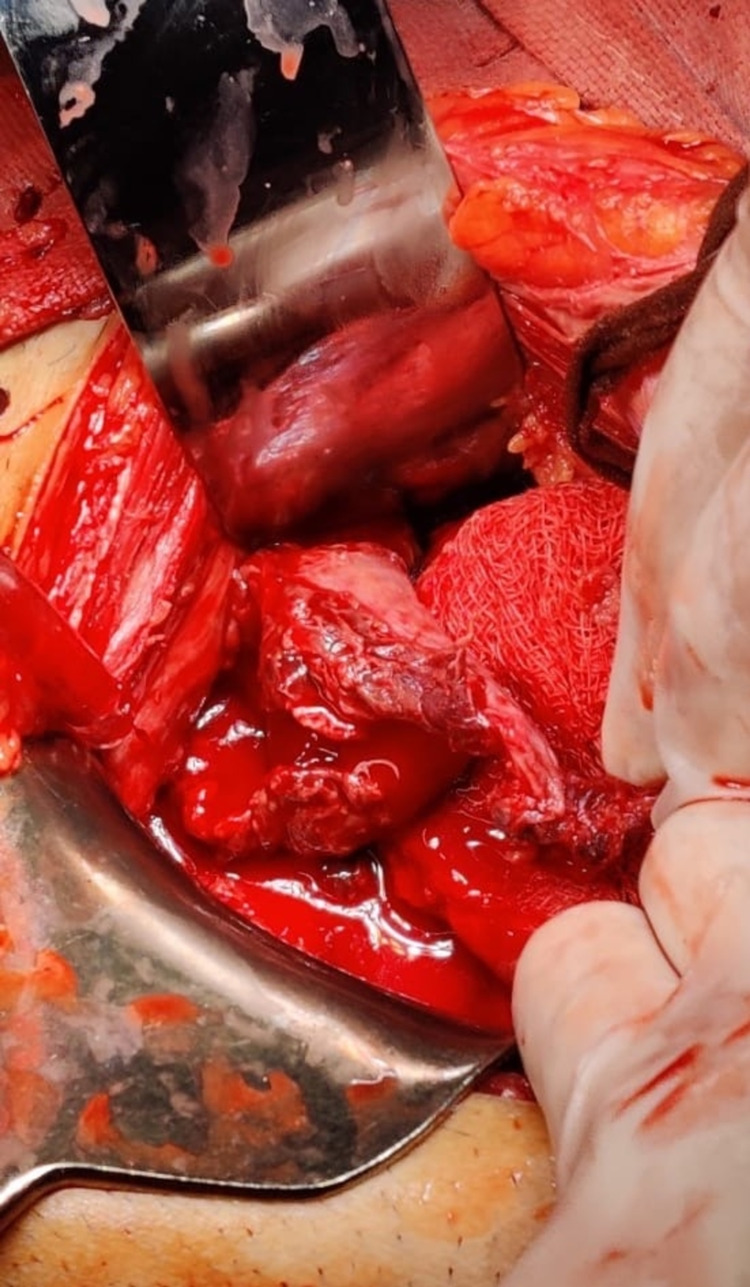
Intra-operative findings showing ruptured rudimentary horn with active bleeding

**Figure 4:**
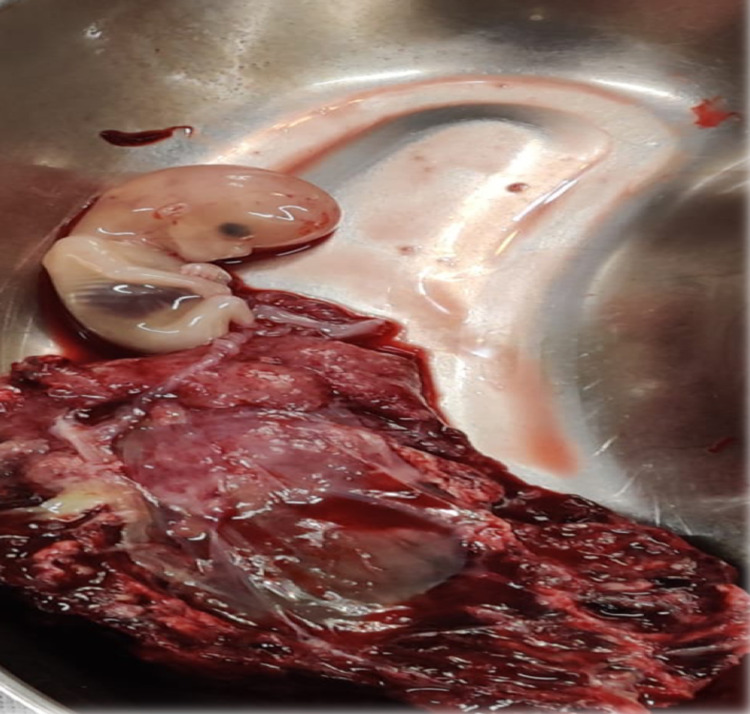
Specimen showing fetus corresponding to 11 weeks period of gestation with placenta

The provisional diagnosis was made of ruptured right rudimentary horn pregnancy and the patient was taken up for emergency laparotomy. Intraoperatively 1.5-liter hemoperitoneum was found with a right ruptured rudimentary horn pregnancy is seen with fetus and placenta lying attached to horn with clots (Figures 3 and 4).

Multiple adhesions were identified between the edematous bowel and anterior abdominal wall seen with necrotic tissue adherent and clumped together as tubo ovarian mass. A rudimentary horn measuring 6x4.5x2 cm was resected. Right tube and ovary not identified. The left part of the uterus, tubes, and ovaries had a normal appearance making the diagnosis of right communicating horn with functioning endometrium. The postoperative course was uneventful and an ultrasound of kidneys, ureters, and bladder showed bilateral normal kidneys with no anomalies. The patient was discharged on post-operative day 4. Histopathology of rudimentary horn specimen showed predominantly hemorrhagic, fibro collagenous tissue with hyalinization, myxoid degeneration, and fatty infiltration along with the presence of chorionic villi, decidualized stroma with endometrial glands, and areas of hemorrhage.

## Discussion

Despite the advancement of various imaging modalities, patients with uterine malformations are still being diagnosed and managed with delay, leading to increased morbidity among women with Mullerian anomalies. As seen in our case, the diagnosis of rudimentary horn pregnancy was missed until 11 weeks gestation and the woman was managed in line of pregnancy with anemia and was diagnosed with ruptured rudimentary horn ectopic pregnancy only after she became hemodynamically unstable.

Congenital uterine anomalies are caused by abnormal formation, fusion, or resorption of Mullerian ducts during the intrauterine period. The overall prevalence of uterine anomalies is approximately 5.8% with 0.1% for unicornuate uterus [1]. Pregnancy in rudimentary horn commonly misdiagnosed due to the presence of myometrium of rudimentary horn around the fetus and usually go up to the second trimester before they present in an emergency with a history of failed induction or with rupture as in our case. Thus early diagnosis and management are essential in reducing, maternal morbidity and mortality associated with this condition. Two-dimensional or three-dimensional ultrasound imaging is the diagnostic modality of choice with a high index of suspicion. Ultrasound criteria for diagnosis of rudimentary horn ectopic pregnancy include (a) pseudopattern of an asymmetrical bicornuate uterus, (b) absent visual continuity between the cervical canal and lumen of the pregnant horn and (c) the presence of myometrial tissue surrounding the gestational sac [2]. Our case fulfilled the second and third criteria.

A review of the literature showed similar cases of misdiagnosed rudimentary horn pregnancies. A case report from Ghana in 2008 showed a case of rudimentary horn pregnancy which was diagnosed before rupture, in a gravida six para five after failed induction for initial diagnosis of missed abortion at a 16-week period of gestation [3]. Another case was reported in 2012 from Oman of primigravida at 22 weeks POG in shock misdiagnosed as abdominal pregnancy with hemoperitoneum, with a final diagnosis of ruptured rudimentary horn pregnancy after laparotomy [4]. Similarly, Jena et al. in 2014 reported a case of ruptured rudimentary horn pregnancy of unicornuate uterus misdiagnosed as abdominal pregnancy presented at 20 weeks with rupture of rudimentary horn managed by resection of the horn [5]. A case report from Karnataka in 2012 by Kanagal et al. showed the second gravida with previous normal vaginal delivery. She was induced at 24 weeks in view of fetal demise; she was initially misdiagnosed as rupture uterus with shock which was later diagnosed with ruptured rudimentary horn pregnancy after laparotomy [6]. A case report in 2018 from Karachi by Hussain et al. showed a primigravida in shock at a 17-week period of gestation initially misdiagnosed as ruptured ectopic pregnancy, which was later diagnosed with ruptured rudimentary horn pregnancy after laparotomy [7]. A recent case report from Haryana in 2020 by Reetu et al. showed a case of ruptured rudimentary horn pregnancy diagnosed after laparotomy for a provisional diagnosis of cesarean scar rupture at 33 weeks of gestation [8]. Thus, the literature review also showed cases of ruptured rudimentary horn pregnancy that are commonly misdiagnosed and usually rupture in the second trimester of pregnancy with life-threatening blood loss with patients presenting in shock, as seen in our case. We report this case as an addition to the already available literature on rudimentary horn pregnancy in which ruptured rudimentary horn pregnancy was missed and managed in line with intrauterine pregnancy with severe anemia.

## Conclusions

Despite advances in ultrasound and other diagnostic modalities, such cases of rudimentary horn pregnancy are still delayed in diagnosis and can lead to complications, including rupture. The diagnosis can be missed in ultrasound especially in inexperienced hands. Thus, a high index of clinical suspicion is needed to reduce morbidity and mortality. There is a need for an increased awareness of this condition especially in developing countries where the possibility of detection before pregnancy or before the rupture is less likely.
